# Institutionalising community participation in decision-making in maternal and newborn health services in low-and middle-income countries: An analysis from 102 national health ministries

**DOI:** 10.1371/journal.pgph.0005139

**Published:** 2025-09-02

**Authors:** Olive Cocoman, Debra Jackson, Harriet Ruysen, Brynne Gilmore

**Affiliations:** 1 Department of Infectious Disease Epidemiology and International Health, London School of Hygiene & Tropical Medicine, London, United Kingdom; 2 School of Public Health, University of the Western Cape, South Africa,; 3 School of Nursing, Midwifery and Health Systems, University College Dublin, Dublin, Republic of Ireland; University of Cape Town, SOUTH AFRICA

## Abstract

In 2024, 194 countries endorsed World Health Assembly Resolution (WHA77.2) to strengthen participation in health-related decision-making. Achieving this requires strong leadership to institutionalise community participation by embedding it into health system functions. However, efforts are often fragmented and short-term, hindering both sustainability and scalability. There is limited understanding of how well countries have institutionalised community participation in decision-making for quality maternal and newborn health services. A secondary analysis of maternal and newborn health survey data was conducted using responses from 102 Ministries of Health in low-and middle-income countries. The analysis assessed progress in adopting and implementing maternal and newborn health recommendations on community participation. A descriptive approach was used to summarise the frequency of reported community participation activities. Percentages were applied to describe the data, which was disaggregated by 2024–2025 World Bank classifications for income level, and fragile and conflict-affected settings*.* Country responses were categorised using Lasswell’s Policy Cycle heuristic. The findings indicate substantial gaps in institutionalising community participation in maternal and newborn health. Only half of countries reported integrating participation into national plans, and just one-third into implementation. In 90% of countries, parent groups were reported to be either absent or lacking influence on policymaking. National research on community participation, essential for evidence-based decision-making, was rarely reported. Across all regions, countries had varied progress, reflecting a diverse and uneven landscape of community participation. Stronger efforts are required to institutionalise community participation across the maternal and newborn health policy cycle. Strengthening this integration will require clear metrics to track implementation, enabling more accurate assessments of progress and accountability. Identifying countries where institutionalisation is advancing can surface positive deviance cases. Studying these in-depth may reveal drivers and effective strategies for fostering community participation to guide the adaption and integration of successful approaches into national health systems.

## Introduction

Community participation is widely advocated for as a key strategy to accelerate progress toward achieving the Sustainable Development Goal (SDG) targets and Universal Health Coverage (UHC) with quality care [1–3]. In 2024, 194 countries endorsed World Health Assembly Resolution 77.2, committing to implement, strengthen, and sustain regular and meaningful participation in health-related decisions across the system at all levels, and to create a safe and enabling environment for participation [4]. However, there is a limited understanding of how well countries have institutionalised community participation in decision-making for health.

### Role of community participation in health system improvement

Health systems provide the foundations to deliver quality care. They are not singular entities, but complex and adaptive-dynamic networks of interdependent components whose behaviour cannot be reduced to the sum of their parts [5]. While difficult to change, health systems must evolve to be continuously learning systems [6]. Learning health systems refers to the development of insights, knowledge, and associations between past actions, the effectiveness of those actions and future actions, and occurs at all levels of the health system [6]. Community participation (or related terminology, as outlined in Box 1), is recognised as vital in such systems. Community input surfaces blind spots, ensures services are user-centred, and improves service uptake - especially when perceived or experienced poor-quality care deters use [7,8]. When community members are involved in decision-making processes, health systems planning becomes more inclusive of community realities, including the social determinants of health, patients’ experiences of care, and the preferences of the population served [3].

Two key findings from implementing community participation are, firstly, health workers and decision-makers become more effective, accountable, and responsive, while community members feel more empowered to interact with health providers and decision-makers [9–11]. Secondly, the ability to implement, sustain, and scale up community participation depends heavily on supportive policy, programmatic, and structural elements. These elements must address persistent challenges such as fragmentation, distrust, hierarchical attitudes among health workers or management, and exclusionary power dynamics [9,12–15]. Amidst the sharp decline in overseas development assistance to many low- and middle-income countries (LMICs), community participation can serve as a valuable prioritisation mechanism, helping identify high-impact barriers to quality care and uncover blind-spots in health systems’ resilience, ensuring that limited investments are targeted and efficient. Leveraging local knowledge is increasingly crucial to strengthen resilience, prioritise critical actions, and ensure responses are grounded in community realities.

**Box 1. Participation terminology**

The literature contains multiple terms that are used interchangeably to describe the involvement of community members with public health institutions in decision-making, such as community participation, community engagement, community involvement, community or social mobilisation, social participation, and social accountability [16]. The preference of terminology has changed over time [16]. Since the 1970s, community participation has been continuously used to describe the participation of community members in health policy, planning, and implementation. It was identified as an integral component of better health outcomes, as set out in the primary health care approach in the Alma-Ata Declaration in 1978. Therefore, this paper uses community participation with its rich history and clear vision of participation as a key intervention to allow service users and community members to actively participate in decision-making regarding the care they receive, and regarding how to ensure access to and delivery of quality care for all [17].

### Institutionalising community participation in health system functions

Institutionalisation is defined as clusters of norms or behaviours with strong but variable mechanisms of support and enforcement [18]. Although the concept of institutionalisation is widely used in health system strengthening, few empirical studies examine how it unfolds in practice. One study highlights institutionalisation as a process that requires capacity building and coordination to stabilise a norm or practice at the district level [19]. Another study describes institutionalisation as an outcome, evidenced by routine, integration, and sustainable practice [19,20]. This paper explores the extent to which community participation is institutionalised - routine, integrated, and sustained - by examining how countries report its incorporation across policy cycles.

Strong health systems rely on the continuous improvement of policies and programmes through iterative cycles [21–23]. These cycles encompass situational analysis, priority setting, planning, budgeting, implementation, monitoring and evaluation (M&E), and review at the facility, sub-national, and national levels [24]. Institutionalising community participation requires leadership at every level to embed community participation in policy development, planning and review processes, and frontline implementation. Moreover, it is equally critical to ensure that feedback and learning from community participation are systematically integrated into national strategies and used to refine implementation. This fosters ownership reduces fragmentation and strengthens national policies and strategies [25].

Achieving this institutionalisation demands investments in multiple health system building blocks. Leadership and governance are fundamental to enabling community participation at all stages of the policy cycle. At the point of care, strong facility leadership is essential to establish and sustain community participation in quality-of-care processes. This in turn must be backed by consistent support from higher-level leadership to ensure the presence of policies, strategies, and structures, and establish clear lines of accountability. These elements are essential to stabilise engagement processes.

Additionally, information systems inform regular evaluation and documentation and are crucial for feeding lessons into policy, planning, and implementation. Dedicated financing is required to resource participation mechanisms and remove barriers to participation. Finally, investments in developing and motivating a workforce that can actively support engagement activity are vital [1,26].

### Gaps in the literature and practice

In reproductive, maternal, newborn, and child health (RMNCH), there is limited data and literature that takes a health system-building perspective necessary to track the institutionalisation of community participation across countries [16]. A recent scoping review on community participation in RMNCAH found that research is heavily concentrated in twelve countries and fifteen donor-supported projects [16]. This dominates the literature with a project lens that fails to reflect how community participation is institutionalised in health system structures.

Beyond the dataset used in this paper, there is no further dataset known on the institutionalisation of community participation in sexual, reproductive, maternal, newborn, child, and adolescent health (SRMNCAH) service delivery. The only known data points on progress for community participation in RMNCH were captured in the World Health Organization (WHO) SRMNCAH Policy Survey conducted in 2018–2019, which included two elements; 1) Are civil society organisations typically included in the SRMNCAH coordinating body (63% of reporting countries responded positively), and 2) Do civil society organisations participate in reviews of SRMNCAH plan (58% of reporting countries responded positively) [27]. However, the most recent SRMCAH Policy Survey conducted in 2023 did not collect any data related to participation [28].

Multiple systematic reviews have concluded that there is a small but significant amount of evidence demonstrating an association between community participation and improved RMNCH [17–24]. The benefits of participation have been noted in design and planning, implementation and delivery, monitoring and accountability, and for building relations between clients[15]. Existing studies on community participation predominantly focus on isolated engagement activities at the point of service, or pilot studies, without detailing the design, content, or the actors involved, or how the activity is linked to national or sub-national strategies [9–12,15,16,29–31]. Even when participation is prioritised in RMNCH policy cycles, little research has explored how the agenda has evolved or how the implementation is sustained and scaled up to inform adaptation or replication in further contexts [26,32,33]. Documented examples of institutionalising community participation through iterative policy cycles - covering planning, implementation, M&E, and review - are rare and appear to be found mostly in the grey literature [34,35]. Even existing guidance that emphasises the importance of embedding community participation in policy cycles, highlights isolated tools or mechanisms and draws from a very small number of countries [1,36]. When viewed through a policy cycle lens, it fails to demonstrate where and how institutionalisation has progressed through repeated policy cycles and what lessons have been learned from doing so. This gap is particularly evident in LMIC settings, where learning from others’ experiences is particularly valuable due to the high burden of mortality, and significant resource constraints. This paper aims to contribute to this gap by examining the status of institutionalising community participation in decision-making for quality maternal and newborn health (MNH) services, using self-reported data from the national leadership in LMICs.

This analysis does not attend to the scope or quality of reported community participation activities - elements that are essential for understanding the true extent of institutionalisation in each country. Empirical evidence indicates that simply increasing participation mechanisms by creating more committees, councils, or report cards, does not necessarily lead to more democratic, transparent, or accountable governance. Participation is vulnerable to elite capture and persistent hierarchies and may be tokenistic, employed to secure funding, fulfill policy requirements, or legitimise top-down decisions rather than foster real power-sharing [9,14,37–39]. This analysis also does not consider the policy, planning and implementation context, which is known to shape outputs and outcomes and is the focus of a subsequent study.

This study is limited to documenting the current status of institutionalisation by examining how countries report embedding community participation in MNH policy cycles. This forms part of a crucial first step in establishing a baseline to assess future progress to meet Resolution 77.2 and identifying existing gaps. In addition, highlighting countries where institutionalisation is reported to be more advanced can help to surface positive deviance cases that warrant deeper analysis to uncover effective strategies for fostering community participation. These insights can inform efforts to scale and adapt successful approaches in other settings.

## Methods

This study involved a secondary analysis of data from an MNH survey completed by 102 Ministries of Health in LMICs. The survey was developed by the WHO, United Nations Children’s Fund (UNICEF), and the United Nations Populations Fund (UNFPA) to monitor the implementation of the Every Newborn Action Plan (ENAP, 2014) [22] and Strategies toward ending preventable maternal mortality (EPMM, 2015) [21,40]. Known as the ENAP EPMM Tracking Tool, the survey aimed to track countries’ progress in adopting WHO MNH recommendations in national health plans, strategies, and guidelines as well as implementation progress. The intent was to stimulate country-level dialogue on implementation gaps and enable comparative and thematic global progress analysis.

### Ethics statement

The London School of Hygiene & Tropical Medicine reviewed and granted an exemption to undertake a secondary analysis of this dataset (LSHTM Ethical Approval REF 31079). Formal permission to use this dataset was provided by WHO and UNICEF, and the data were used in accordance with the agreed terms and conditions. To mitigate the risk of misinterpretation or misrepresentation, the data have been analysed within the context of their original purpose and known limitations. The dataset contains no personally identifiable information and is stored securely on password-protected systems and accessible only by authorised team members involved in this research. The authors bear sole responsibility for the analysis and the interpretations presented.

### Data source

The most recent ENAP EPMM survey was distributed via email to 106 WHO Member States at the end of 2022. UNICEF Country Offices communicated the survey to the relevant Ministry of Health RMNCH/MNH Technical Working Group (TWG) or equivalent, with an indicated time-frame for completion. Country responses to the survey were validated through follow-up by UNICEF and WHO in 2023. We obtained the secondary data from WHO and UNICEF, which set out the original country responses to all questions and national documents submitted to validate and substantiate the country’s responses.

The ENAP EPMM survey was modular with components for various stages of MNH service delivery, and thematic areas aligned with the health systems’ building blocks. It consisted of 124 questions and an additional 29 sub-questions. For this study, the authors reviewed all questions and selected those relevant to community participation. This included; all questions and responses that explicitly referred to community participation or related terminologies such as engagement or mobilisation, including questions related to activities to provide participatory spaces for community representatives, such as Maternal and Perinatal Death Surveillance and Response (MPDSR) programmes. Secondly, questions related to community health workers (CHW) were included because this particular health cadre is embedded in families, communities and the health system and is frequently put forward as accountable for community participation activities [41,42]. Further, two open-ended questions on investments in research and innovation were included where responses included community participation. A total of thirteen questions were identified through this process. Table 1 sets out these 13 questions.

**Table 1:** Questions on community participation included in the ENAP EPMM Tracking Tool.

Planning	*Questions 1, 2 & 3:* National RMNCH or relevant MNH plan factors in community participation in MNH services			*Questions 4 & 5:* National Quality of Care plan (relevant for MNH) sets out community participation		*Question 6:* Human Resources plan ensures provision for community health workers (CHW) (paid/unpaid)	*Question 7:* National level: representation from civil society, women’s, or parent groups in the RMNCH/MNH TWG
	In defining priorities	In planning	In M&E	In defining priorities & planning	In M&E		
Implementation	*Question 8:* District/Facility level mechanism for community participation			*Question 9:* MPDSR facility review process includes community stakeholders		*Question 10:* Core competencies defined for community health or extension workers	*Question 11:* Innovations between 2020-2022
M&E	*Question 12*: Research Investments 2020-2022						
Review	*Question 13:* Parent groups for MNH &/or stillbirth prevention influence government policy through citizen-generated data						

### Analysis approach

A descriptive analysis was conducted to summarise how frequently community participation was reported. Percentages were applied to describe the data, which was disaggregated according to the 2024–2025 World Bank classifications for LMICs as well as fragile and conflict-affected settings [43]. Four of the thirteen questions (1, 2, 3, and 9 in Table 1) were previously reported on at the aggregate global level in the MNH Progress Report in 2023 and had not been analysed at country or region levels [44]. For this analysis, Microsoft Excel was used to generate the tables and figures.

To better understand the policy context of reported community participation activities, country responses were categorised using Lasswell’s Policy Cycle heuristic. This is a heuristic tool first developed by Lasswell (1956) to simplify the complexity of policy development and implementation by separating the different stages of the policy cycle [45]. This cycle is applicable at facility, district, subnational, and national levels [24]. In this data set, the national policy cycle stages were represented as follows: planning (7 questions), implementation (4 questions), monitoring and evaluation (1 question), and policy review (1 question).

## Results

Of the 131 LMICs classified by the World Bank for 2024–2025, 102 voluntarily completed the ENAP EPMM Survey responding to the thirteen selected questions. The findings illustrate the variation in the extent and nature of efforts to institutionalise community participation in MNH services.

### 1. Gaps along the national policy cycle

**Fig 1:**
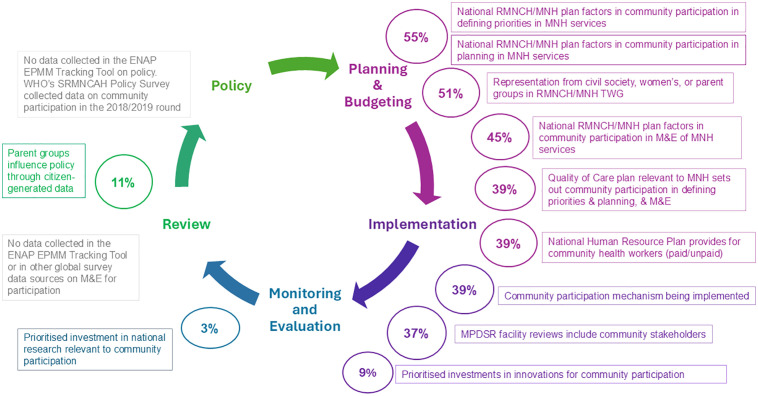
Community participation in maternal and newborn health: reported engagement across the national policy cycle stages in 102 LMICs.

The analysis showed a progressive decline in the reporting of community participation across the stages of the policy cycle. While more responses described community involvement during the planning phase - especially in national RMNCH/MNH strategies - fewer countries reported participation in later stages such as implementation, monitoring and evaluation, and policy review. This trend was observed despite differences in the number of questions assigned to each stage. Fig 1 illustrates the decline in reported community participation across the policy cycle stages.

#### 1.1. Gaps in national planning.

In 2023, approximately half the LMICs reported that their national RMNCH/MNH plans incorporated community participation to help define priorities (55%, n = 56) and support planning processes (51%, n = 52). However, fewer national plans included community participation in M&E activities, indicating a gap in ensuring participation in assessing the effectiveness and quality of MNH services (45%, n = 46). For Quality-of-Care plans related to MNH, community participation was reported at an even lower rate (39%, n = 40).

#### 1.2. Gaps in participatory spaces for decision-making.

Across LMICs, the reported provision of participatory spaces for community involvement in MNH decision-making remained limited (See Table 2 and S1 Table). A participatory space refers to a physical or virtual setting where people come together to interact and contribute to decision-making processes [3].In 2023, 51% (n = 52) of LMICs reported providing a participatory space for community representation from civil society, women's groups, or parent advocacy groups in the RMNCH/MNH TWG in the Ministry of Health. Only 19% of LMICs reported having active parent groups (n = 20). Of these, just half reported that parent groups influence government policymaking through the use of citizen-generated data (n = 11*;* Armenia, India, Kenya, Malawi, Mali, Moldova, Montenegro, Serbia, Sudan, Tuvalu, and Uganda). At the facility level, 63% (n = 65) reported that community participation in MPDSR processes was absent.

**Table 2:** Summary of reported community participation in maternal and newborn health by WHO Region and fragility classification (n = 102).

REGION (number of countries reporting / number of LMICs in the region)	A. Establish community participation in national plans					B. Provide participatory spaces for community representatives/members in decision-making with health authorities				C. Foster community health worker cadre		D. Community participation cited as a top 3 MNH investment	
	National RMNCAH or relevant MNH plan factors in community participation in MNH services			National MNH Quality of Care plans set out community participation		6. At national level: representation from civil society, women’s or parent groups in the RMNCH or MNH TWG	7. Parents groups for MNH &/or stillbirth prevention influence government policy through citizen-generated data	8. District /Facility level mechanism provided	9. MPDSR facility review process includes community stakeholders	10. HR plan ensures provisions for community health workers (paid/unpaid)	11. Core competencies defined for community health or extension workers	12. Research investment between 2020-2022	13. Innovation investment between 2020-2022
	1. In defining priorities	2. In plan-ning	3. In M&E	4. In defining priorities & planning	5. In M&E								
Sub-Saharan Africa: 44 of 47 LMICS reported													
% of 44 LMICS	77	77	73	59	61	70	11	57	50	48	84	1	7
South Asia: 8 of 8 LMICS reported													
% of 8 LMICS	75	50	38	25	38	63	13	38	50	50	63	0	2
Middle East & North Africa: 12 or 13 LMICS reported													
% of 12 LMICS	42	33	17	25	17	33	0	25	25	17	42	0	0
East Asia & Pacific: 17 of 22 LMICs reported													
% of 18 LMICS	24	24	18	24	12	18	6	18	18	18	29	0	0
Europe & Central Asia: 13 of 18 LMICs reported													
% of 13 LMICS	31	31	15	15	8	38	31	23	8	0	23	1	0
Latin America & Caribbean: 8 of 23 LMICS reported													
% of 8 LMICS	38	25	50	38	63	50	0	38	63	50	63	0	0
GLOBAL TOTAL: 102 of 131 LMICS reported													
% of 102 LMICS reporting	55	51	45	39	39	51	11	39	37	33	59	3	9
FRAGILE AND CONFLICT SETTINGS (FCS): 36 of 39 reported													
% of 36 FCS	61	50	44	33	33	50	8	31	36	31	64	0	0

**Fig 2:**
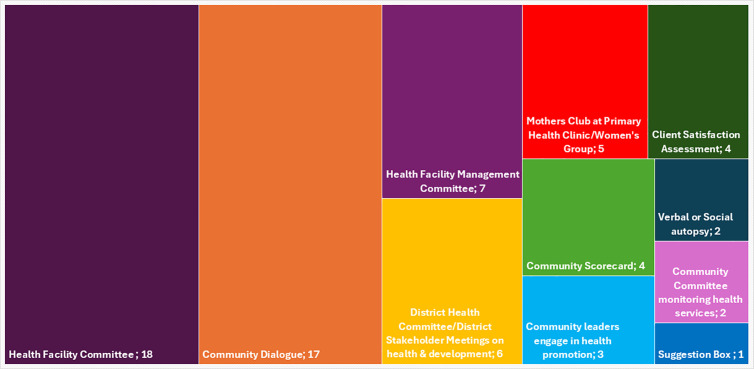
Reported mechanisms for community participation in maternal and newborn health decision-making at district and facility levels in 40 LMICs.

Fig 2 shows the reported participatory mechanisms and their frequency. Only forty countries identified one mechanism to engage community members, and just seventeen countries reported using more than one. The most frequently cited mechanisms were facility level health committees (n = 27) named as health post committees (n = 18) and health facility management committees (7).Secondly, community dialogues were commonly reported (n = 17), and thirdly, district-level health committees (n = 2) and stakeholder meetings (n = 4). Finally a few countries reported client satisfaction surveys (n = 4), and community scorecards (n = 4) or community committees to monitor results (n = 2). Some mechanisms extended beyond MNH, such as health and sanitation committees (India) and public participatory planning (Musrenbang, Indonesia).

#### 1.3. Role of community health workers (CHW).

Only 33% (n = 34) of LMICs reported that National Human Resource Plans include provision for CHWs, whether paid or unpaid. However, more countries (n = 60) reported to have defined CHW core competencies as public health professionals. Where implementation mechanisms were reported, countries commonly responded positively to both CHW-related questions (Table 2).

#### 1.4. Investment in innovation or research.

Few countries reported investing in innovations or research related to community participation (n = 9) (Table 2 and S1 Table). Regarding innovations, Ghana specified their community scorecard systems for quality improvement, Tanzania noted the nationwide rollout of the digital client feedback system (Mama na Mwana), and Uganda cited Family Connect, an SMS-based tool for women to rate the health services received. Iraq highlighted the completion of the first comprehensive national communication strategy and operational plan for 2022–2027, which includes support for active community participation in MNH. In response to an additional open-ended question about the top-three national research investments, only two countries referenced community participation; Ghana cited research on women’s experience of care during childbirth, and Serbia cited research on family-orientated neonatal care.

### 2. Regional variation in community participation in maternal and newborn health

All regions included countries at varying stages of advancing community participation, reflecting a highly diverse landscape. The greatest involvement was reported in the Sub-Saharan Africa and South Asia regions, and the least was in East Asia and the Pacific Region (Tables 2 and 3).

**Table 3:** Range of reported progress across 102 countries, by World Health Organization Region.

Region	Yes, to 11 + questions	Yes, to 10 questions	Answered yes to 2–9 questions (in descending order)	Report yes to only 1 question	No to all 13 questions
Sub-Saharan Africa (44/47) *Fragile and conflict setting	Ghana, Malawi, Sudan*, Tanzania, Uganda **(Total: 5)**	*Parents’ groups do not exist or influence government policymaking:* Democratic Republic of Congo*, Ethiopia*, Gambia, Guinea, Rwanda, Lesotho, Togo, Zambia (8) *Human Resources plan has provisions for CHWs:* Mali*, Kenya (2) **(Total: 10)**	9: Benin, Burundi*, Cabo Verde, Cameroon*, Liberia, Nigeria*, South Sudan* (7) 8: Central African Republic*, Madagascar, Senegal, Sierra Leone (4) 6: Burkina Faso*, Chad*, Eritrea*, Niger* (4)	Community representative on TWG: Mauritania (1) Defined CHW core competencies: Equatorial Guinea, Guinea Bissau*, Somalia* (3) Human Resources plan has provisions for CHWs; Gabon (1) **(Total:5)**	Congo*, Namibia, São Tomé and Príncipe * (Total: 3)
			5: Cote D’Ivoire, Eswatini (2) 4: Mozambique* (1), 3: Comoros* (1) 2: Angola, Zimbabwe*(2) **(Total: 21)**		
**South Asia (8/8)**	India **(Total: 1)**		8: Pakistan (1), 6: Bangladesh (1)		Bhutan **(Total: 1)**
			5: Nepal, Maldives (2) 3: Afghanistan*, Sri Lanka (2) **(Total: 6)**		
**East Asia & Pacific (17/22)**	(0)		7: Mongolia (1), 6: Tuvalu* (1)	*Community involvement in MPDSR*: Marshall Islands*, Timor Leste* (2) *Human Resources plan has provisions for CHWs*: China (1) *Defined CHW core competencies*: Vietnam (1) **(Total:4)**	Fiji, Kiribati*, Micronesia*, Samoa, Solomon Islands*, Tonga, Vanuatu **(Total: 7)**
			5: Cambodia, Lao PDR, Papua New Guinea* (3) 3: Indonesia (1) **(Total: 6)**		
**Middle East and North Africa (12/13)**	(0)		9: Iran (1), 8: Jordan (1), 6: Morocco (1)	*Community representative on TWG:* Syria*, Palestine* (2) *Quality of Care plan for MNH plan sets out community participation in M&E:* Egypt (1) **(Total: 3)**	Djibouti, Iraq*, Libya*, Tunisia **(Total: 4)**
			4: Yemen* (1) 2: Lebanon* (1) **(Total: 5)**		
**Latin America & Caribbean (8/23)**	(0)	*Parents’ groups do not exist*: Cuba (1) **(Total: 1)**	6: Haiti*, Paraguay (2)	*Community representative on TWG*: Argentina (1) *National MNH plan factors in participation in M&E:* Bolivia (1) **(Total: 2)**	(0)
			3: Dominican Republic, Nicaragua (2) 2: Guatemala (1) **(Total: 5)**		
**Europe and Central Asia (13/18)**	(0)		6/11: Armenia, Kazakhstan (2)	*Defined CHW core competencies*: Azerbaijan (1) *Human Resources plan has provisions for CHWs:* Turkmenistan (1) *Parents’ groups exist/ influence in government policymaking*: Montenegro: Kyrgyz Republic (2) **(Total: 4)**	Georgia, North Macedonia, Uzbekistan **(Total: 3)**
			5: Moldova, Tajikistan (2) 3: Albania, Serbia (2) **(Total: 6)**		
**TOTAL (n = 102/131)**	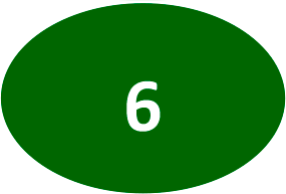	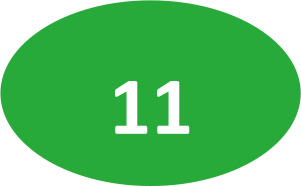	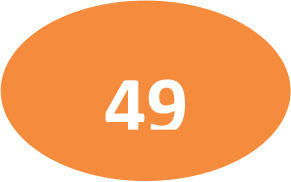	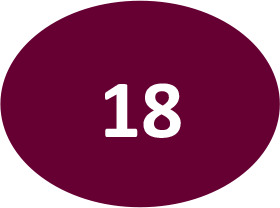	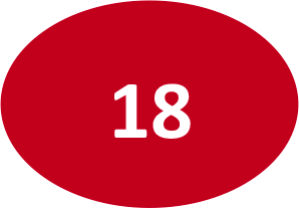

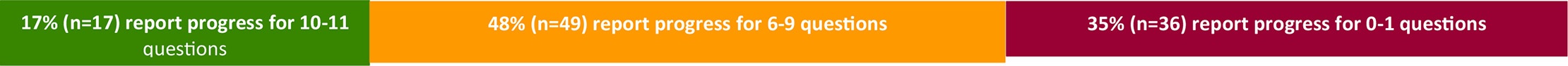

#### 2.1. One-third of countries report no or minimal community participation.

Overall, in 2023, one-third (n = 36) of the 102 LMICs included in this study reported community participation was absent on the MNH agenda; 17% (n = 18) reported no to all 13 survey questions, and a further 17% (n = 18) of countries answered yes to only one survey question.

#### 2.2. Most progress reported in seventeen countries.

No country reported positively to all 13 questions or 12 questions. However, one-sixth (n = 17) responded positively to 10 or more questions. A very small subset of these countries (n = 6), responded positively to 11 of 13 survey questions; Ghana, India, Malawi, Sudan, Tanzania, and Uganda. The additional eleven countries that reported positively to 10 questions are; Cuba, the Democratic Republic of Congo, Ethiopia, Gambia, Guinea, Kenya, Mali, Rwanda, Lesotho, Togo, and Zambia. Among this group of seventeen, two questions were consistently missing; 1) whether parent groups influence policymaking through citizen-generated data, and 2) whether the country had invested in research or innovation related to community participation. Notably, most countries reporting positively to the majority of questions are in Sub-Saharan Africa (Table 3).

#### 2.3. Significant regional variation in the reported action to ensure community participation in MNH services.

Table 2 provides a breakdown per WHO region. Each country’s data is set out in S1 Table.

### Sub-Saharan Africa

Data are available for 94% of LMICs (n = 44/47). The region stands out for its strong commitment to community participation in MNH; one-third of LMICs (n = 15) answered yes to 10 or more survey questions. Only three LMICs - Congo, Namibia, and São Tomé and Príncipe - responded no to all questions. Three-quarters reported incorporating community participation in national MNH plans for defining priorities, planning (77%, n = 34), and M&E in MNH services (73%, n = 32). Over 70% (n = 31) reported civil society participation in national MNH TWGs, and 60% (n = 27) reported participation in quality-of-care planning.

However, fewer countries reported implementation at district and facility levels. Just over half of Sub-Saharan African LMICs reported on engagement mechanisms; primarily referencing health committees (n = 15), community dialogues (n = 12), and community scorecards (n = 4). Sub-Saharan Africa also leads in fostering a CHW cadre; half of LMICs (n = 21) reported ensuring provision for CHWs in MNH Human Resource Plans, and 84% (n = 37) reported defining CHW core competencies.

### South Asia

Data are available for 100% of LMICs (n = 8/8). India and Pakistan emerged as the most active countries in the region with positive responses to most questions. Three-quarters of LMICs (n = 6) reported incorporating community participation in defining MNH priorities. However, involvement declined in planning (n = 4) and M&E activities (n = 3). Only Bangladesh, India, and Pakistan reported that community participation is factored into quality-of-care plans and provided examples of implementation. Nearly two-thirds (n = 5) reported representation from civil society, women’s groups, or parent advocacy groups in national RMNCAH/MNH technical working groups, but only India noted that parent groups influence government policy. Half of the countries reported community involvement in MPDSR activities.

### Middle East and North Africa

Data are available for 92% of LMICs (n = 12/13). Community participation is reported to be weak in the region. One-third of the countries (n = 4; Djibouti, Iraq, Libya, and Tunisia) reported no activity for all questions. The most frequently reported activities are community participation in planning (n = 4) and representation in TWGs (n = 4). Three countries (Jordan, Morocco, and Iraq) reported community involvement in MPDSR and also provided implementation mechanisms to engage communities; Jordan cited community dialogues, and Morocco highlighted planning and verbal autopsies. Iraq reported a new comprehensive communication strategy and operational plan (2022–2027) that includes community participation.

### East Asia and the Pacific

Data are available for 77% of LMICs (n = 17/22). There is a low level of reported activity. Half the region’s LMICs (n = 11) reported no activity in responses to 10 or more questions. The most commonly reported activities include factoring in community participation in RMNCH/MNH plans or quality of care plans in defining priorities and planning (n = 4; Indonesia, Lao PDR, Mongolia, and Tuvalu) and ensuring Human Resource plans provide for CHW (n = 4; China, Lao PDR, Mongolia, and Tuvalu). Three countries (Cambodia, Papua New Guinea, and Tuvalu) reported representation from civil society, parents, or women’s groups in the national RMNCH/MNH TWGs, and only Tuvalu reported parent groups influence government policies. Cambodia, Marshall Islands, and Timor-Leste reported community involvement in MPDSR. Three LMICs exemplified participation mechanisms (Cambodia, Indonesia, and Mongolia).

### European and Central Asia

Data are available for 72% of LMICs (n = 13/18) and reveal a low degree of community participation. Over half (n = 7) reported no for 10 or more questions, accounting for one-third of all LMICs in the region. Only Armenia and Kazakhstan reported including community participation in quality-of-care plans. The most frequently reported action was representation from civil society, women’s groups, or parent groups in MNH technical working groups (n = 5/13). Notably, one-third of LMICs (Armenia, Moldova, Montenegro, and Tajikistan) reported active parent groups that influenced government policies through citizen-generated data, one of the strongest findings globally.

### Latin America and the Caribbean

Only 35% (n = 8/23) of LMICs submitted the survey. Among these, half (n = 4) reported community participation in MNH/RMNCH TWGs (Argentina, Cuba, Guatemala, Haiti), in MPSDR (Cuba, Dominican Republic, Guatemala and Paraguay), as well as providing for CHW in national HR plans and defining CHW core competencies (Cuba, Dominican Republic, Haiti and Paraguay).

#### 2.4. Community participation in MNH reported in fragile and conflict-affected settings.

The data set covers thirty-six of the thirty-nine Fragile and Conflict Settings (FCS) classified by the World Bank (2024–2025) (denoted with an asterisk in Table 3 and S1 Table). One-third of all FCS report no community participation activity (n = 13); Congo, Iraq, Kiribati, Libya, Marshall Islands, Micronesia, the State of Palestine, São Tomé and Príncipe, Solomon Islands, Somalia, Syria, Timor-Leste, and Zimbabwe. Around 60% (n = 22) report that community participation was factored into national RMNCH/MNH plans to define priorities in MNH services, and 50% (n = 18) reported participation in MNH health service planning. Similar to the findings for all countries, the reported involvement was less in M&E (44%) (n = 16) and in the implementation stage; just 30% (n = 11) named a mechanism, and one-third reported community involvement in MPDSR (36%) (n = 13). Iraq highlighted future plans, as noted at at 2.3. 

## Discussion

In 2024, 194 countries endorsed World Health Assembly Resolution 77.2 affirming the need to institutionalise community participation in health decision-making. While the question of how to advance community participation is not new, it gains renewed significance in the face of cuts to overseas development assistance (ODA), ongoing pandemic threats, and the pressing need to reduce mortality and improve health outcomes for millions of women and children. In the aftermath of Ebola and COVID-19, where the value of community participation was emphasised, there is a heightened call to better understand how to create an enabling environment for engaging communities in the processes of discussing, designing, delivering, measuring, and resourcing health care. Documenting the current status across countries is an essential first step to assessing progress, and identifying the technical expertise and resources needed to further institutionalise practices. Based on this analysis, five key findings emerge.

### Limited participation in decision-making for MNH services in most LMICs

Globally, across LMICs, there is a distinct lack of community involvement in decision-making for MNH services. This analysis highlights a critical gap in developing and implementing a national vision and strategy to embed community participation in decision-making processes. One-third of LMICs (n = 36) reported no activity related to community participation across the policy cycle. These countries span all regions, with the highest concentration in East Asia and the Pacific regions, and the Middle East and North Africa. Many are fragile settings where community participation is critical to ensure that health systems remain responsive and resilient to both current and emerging community needs [46].

### A significant gap between national planning and implementation

A major disconnect exists between national planning and ground-level implementation. The reported inconsistent emphasis across the stages of the policy cycle raises concerns about the strength of leadership in executing national strategies for community participation in MNH services. While community participation is most often considered during the initial policy cycle stages - such as in defining priorities (55%) and planning (51%) - this focus wanes during implementation. Moreover, few countries reported conducting research on community participation, despite its importance for evidence-based decision-making.

This gap between planning and implementation is evident in the limited reporting of engagement mechanisms. Firstly, only 37% of countries report community involvement in MPDSR. This represents a missed opportunity for community representatives to contribute at multiple stages of the process; to share contextual knowledge, support appropriate quality improvement actions, and ensure accountability of the populations served [47–50].

Secondly, there is a notable lack of reported community participation in health district governance, where critical funding and planning decisions are made closer to communities. Only six countries report district level participation mechanisms including participation in the district reproductive and child health TWG in Mali, local government health committee meetings in Sierra Leone and Bangladesh, and public participatory planning in Indonesia.

Thirdly, community involvement in health facility governance remains limited. Only twenty-five LMICs, mainly in Sub-Saharan Africa, reported the presence of committees at health posts or facilities. This trend may be influenced by the Bamako Initiative Resolution in 1987, which recommended Health Facility Committees (HFCs) as vehicles for community involvement in primary health care. Evidence from systematic reviews suggests that HFCs can improve MNH outcomes by addressing community concerns, supporting health facility management, and holding health workers accountable [9,12,15,51]. Outside Sub-Saharan Africa, only Albania, Cambodia, and Mongolia reported HFC implementation.

Additional mechanisms can bring communities into dialogue with health facilities to improve the quality of care. Community dialogues, a participatory approach to discuss pressing health issues and explore solutions, were reported in seventeen countries; including Bangladesh, Burundi, Central African Republic, Democratic Republic of Congo, Ghana, Jordan, Madagascar, Rwanda, South Sudan, and Togo.

Similarly, community scorecards (CSC) have been shown to enhance transparency in facility operations, responsiveness to community needs, improve health literacy, and foster collective problem-solving [13,29]. Their use has been associated with tangible improvements in MNH health outcomes, including increased health budgets, reduced drug stock-outs, upgraded infrastructure, enhanced water and sanitation facilities, increased provision of beds, and higher antenatal and postnatal attendance rates [13,29,52–59]. Despite their potential, only four LMICs - Ethiopia, Ghana, Kenya, and Malawi - reported using CSCs in MNH.

### The role of the health workforce in enabling participation

Effective community participation requires a well-prepared and supported health workforce. Strengthening the health workforce’s capacity to engage communities demands national-level planning to ensure health workers are adequately trained, compensated, and mentored to engage meaningfully with communities. The data reveals that only one-third of countries include provisions for CHW (whether paid or unpaid) in the National Human Resource Plan, although 60% report having defined CHW core competencies. Countries that reported a supportive planning environment for CHWs were more likely to report community participation activities. This highlights the critical role that CHWs play in community participation. However, CHWs are often tasked with addressing broader health system weaknesses, and their role in promoting accountability should be clarified and strengthened. To address this, all health worker cadres involved in community participation activities need clearly defined roles, and support through national plans and resource allocations [14,42].

### Patient voice in MNH policy generation remains undervalued

The data reveals a widespread undervaluation of the patient or client voice in MNH policy development. Only four LMICs - Cambodia, Lesotho, Pakistan, and Rwanda - reported the use of client satisfaction surveys at the facility level. This is strikingly low, given the potential of such surveys to enhance patient and provider education, inform policy, and improve service delivery and governance [60].

In 90% of LMICs, parent groups were reported to lack influence in policymaking. A notable exception is European LMICs, where nearly half of the countries reported parent groups’ influence on government policy. Globally, this is concerning given the vital role that parents and parent organisations play in advocating for the rights and respectful care of stillborn babies and newborns, particularly preterm infants [61]. Patient advocacy has consistently demonstrated its power to drive political attention, mobilise resources, and improve the quality and coverage of care in areas such as HIV, tuberculosis, and child health [61–63]. Therefore, the absence of formal platforms for patient voices in MNH planning and policy represents a major gap to be addressed in planning and resourcing, as well as in MNH advocacy.

### Geographic variation in community participation

Community participation in MNH decision-making was reported across diverse geographies, with the highest levels of activity in Sub-Saharan Africa and South Asia. Both regions indicate positive steps towards integrating community perspectives into MNH policy and programming.

In Europe and Central Asia, parent groups were more often reported as influencing policy, while in Latin America and the Caribbean, community involvement in MPDSR was more frequently noted. In contrast, in the Middle East and North Africa, and East Asia and Pacific regions, community participation is mostly limited to the planning stage.

Across all regions, several countries reported substantial engagement at both the planning and implementation stages, while others reported minimal or no activity. This variation highlights the importance of understanding the contextual and systemic factors that shape the agenda for community participation in MNH. Identifying and addressing these factors is essential to promoting more inclusive policymaking and implementation globally, and investment in this research is warranted.

### Institutionalising community participation in MNH services: Key Recommendations

In many countries, substantial effort is needed to robustly integrate community participation into health system functions. Analysis of this UN-led data set provides an important opportunity to understand the national-level ambitions around the institutionalisation of community participation in MNH policies and practices. Based on the findings, two key recommendations are proposed.

Firstly, there is a clear need for systematic tracking of progress to institutionalise community participation at each stage of the policy cycle. Secondly, enhanced research efforts should focus on how progress is achieved through investigating contexts, mechanisms, motivations, and impacts of participation along the policy cycle.

1. Systematically track progress to institutionalise community participation

Systematic tracking of progress to institutionalise community participation in MNH is essential to assess whether community participation is being embedded sustainably in MNH systems. Applying a policy cycle lens offers a structured approach to assess institutionalisation, by asking key questions, such as:

- Policy existence: Does a national policy mandate community participation in MNH decision-making?
- Operational planning: Are roles and responsibilities for engagement defined in operational plans?
- Budgeting: Are there dedicated funding lines for engagement activities? Do district and facility budgets incorporate funds to respond to community feedback?
- Implementation evaluation: What mechanisms for community participation have been put in place? Are they functioning effectively? Are they regularly evaluated?
- Monitoring and evaluation: Are M&E findings used to refine policies and programmes?
- Cost-effectiveness: Has the value for money of community participation been assessed?
- Research use: Are studies on barriers and enablers of community participation informing strategies?

Currently, there is no global dataset that captures whether national policies relevant to MNH support community participation. Incorporating such indicators into future rounds of tracking tools like the *ENAP EPMM Tracking Tool* and *SRMNCH Policy Survey* would help address this gap.

In terms of M&E, the ENAP EPMM Tracking Tool has not yet assessed whether countries systematically evaluate the outcomes and effects of community participation policies and programmes. Yet, this activity is essential for making iterative improvements in strategy design and implementation and should be included in future progress tracking. Given the relational nature of engagement - at the individual (micro), groups (meso), and health system (macro) levels - outcomes will differ across each level, and country-level evaluations should attend to all levels [64].

Additionally, institutionalisation requires the identification and monitoring of coordination mechanisms. These mechanisms help embed engagement activities within health systems by stabilising structures, managing risks, and ensuring accountability [26]. WHO’s 2024 *Facilitators’* g*uide for conducting national and subnational programme reviews for maternal, newborn, child and adolescent health* recommends tracking such coordination mechanisms to guide implementation and support progress [65].

Finally, it is equally important to assess the scope and quality of community participation at national, district, and facility levels. The scope, quality, and functionality of all community participation mechanisms vary widely. Clear process and outcome measures should be defined, agreed upon, and routinely tracked. These indicators should intentionally capture not just whether engagement occurs, but whether it is meaningful, inclusive, and leads to improvements. Participation can be shaped by power dynamics and is susceptible to elite capture; therefore, evaluating its quality in context is critical. WHO’s 2021 guidance on *Integrating stakeholder and community engagement for quality MNH outcomes* (2021) details opportunities for engagement along the continuum of engagement. This framework can help to assess whether engagement efforts are moving beyond passive information sharing to genuine collaboration in the co-production of care, where communities shape decisions and outcomes [36].

In summary, adequately resourcing the systematic tracking and evaluation of progress is essential to institutionalising community participation and unlocking its full potential for transforming MNH services.

1. Strengthen implementation research to inform policy and scale-up

Studying countries where institutionalisation is advancing can offer valuable lessons for other contexts, highlighting opportunities for deeper research and cross-country learning. Investing in implementation research and process evaluations is essential to understand what works, for whom, and under what circumstances. These can inform upstream policy responses, strengthen policy formulation, and guide resource allocation. In-depth country case studies, particularly from contexts making notable progress to institutionalise community participation, can serve as concrete exemplars. Such studies can illuminate the complex interplay of systems and the contextual factors that enable or hinder implementation [66].

The current evidence base on institutionalising community participation in RMNCH remains limited. A recent scoping review found that research is heavily concentrated in a small number of countries and donor-supported projects. Since Alma-Ata, the peer and grey literature have been dominated by findings from just twelve countries, reflecting concentrated funding and documentation efforts [16]. Moreover, systematic, scoping, and realist reviews continue to highlight the lack of details regarding how community participation has advanced within national or subnational policy cycles [9,12,15,16,29–31,67].

Notably, one-third of the RMNCH literature describing community participation since Alma-Ata derives from the same fifteen donor-supported projects [16]. This has dominated the literature base with a project lens that fails to reflect how community participation functions are institutionalised in broader health system structures. This reality leads to persistent calls for a richer evidence base that captures the system-wide supportive policies and planning environment, the context, content, purpose, and design of the participation, as well as the roles and motivations of diverse actors and stakeholders involved, and the quality of their engagement [3,16,26,32,33].

The ENAP EPMM Tracking Tool data reveals that more than fifty countries across all regions report active community participation in MNH, responding affirmatively to six or more survey questions. Among these, a small group of seventeen countries reported participation across almost all stages of the policy cycle (Cuba, Democratic Republic of Congo, Ethiopia, Gambia, Ghana, Guinea, India, Kenya, Lesotho, Mali, Malawi, Rwanda, Sudan, Tanzania, Togo, Uganda, and Zambia). One notable example is Ghana. In Ghana, community participation has been integrated into national MNH policy, planning, and quality standards. Following a successful pilot of community scorecards to improve the quality of MNH care, the initiative was scaled up to all sixteen regions. Each region has a Regional Quality Director responsible for overseeing CSC activities, which are embedded into facility assessments and tracked through the national MNH Quality of Care dashboard. By 2023, Ghana had institutionalised this participation mechanism nationwide [61,68,69]. Further, Ghana is the only country in the dataset to report having prioritised research on the experience of care in its national research agenda for 2020–2022. To support broader learning efforts on institutionalising community participation, examples like Ghana should be unpacked, documented, and widely disseminated. These case studies can help bridge the gap between pilot efforts and sustainable, system-level institutionalisation.

Finally, the variation in reported participation across countries underscores the importance of understanding the context-specific drivers of progress. Exploring these drivers, including the technical, political, and financial inputs that enable sustained and scalable participation, can help other countries adapt and embed successful approaches within their health systems.

## Strengths and limitations

Although the dataset is based on self-reported progress by Ministries of Health, it represents a crucial first step in understanding the leadership and actions taken to institutionalise community participation in MNH services across LMICs. Despite the inherent limitations of self-reporting, such as potential biases, inaccuracies, or overstatement of progress, the data nonetheless provide valuable insights into national commitments and efforts to prioritise community participation in MNH.

To minimise the reporting burden on Ministries of Health, and survey fatigue, the ENAP EPMM Tracking Tool (2022–2023) limited the overall number of questions. This design choice, while pragmatic, constrained the depth and scope of data collection, particularly regarding community participation policies, and M&E systems. Furthermore, the tool does not include measures to assess the scope or quality of reported activities - elements that are essential for understanding the true extent of institutionalisation in each country. This gap should be addressed in future rounds of data collection and research. Additionally, inconsistencies in question framing may have led to misunderstanding or uneven responses. For example, within RMNCH/MNH planning, three distinct questions were asked about defining priorities, planning, and M&E. In contrast, the section on quality-of-care planning combined the first two into a single question. Such discrepancies may affect how countries report their activities and should be considered in future tool revisions to ensure greater clarity and comparability across thematic areas.

A notable strength of this dataset is its extensive coverage across nearly all countries in three high-burden regions - Sub-Saharan Africa, South Asia, the Middle East and North Africa as well as a substantial number of countries in East Asia and the Pacific, and Europe and Central Asia Regions. Ministries of Health from 102 countries contributed data across 13 key indicators enabling global analysis of progress and gaps in institutionalising community participation. This broad geographic coverage provides a meaningful foundation for assessing national-level leadership in high-burden settings. However, the dataset could be enhanced by including all LMICs and adding survey questions on M&E and review of community participation, resource allocation, and coordination mechanisms which are critical elements both for implementation and sustainability of participation efforts.

## Conclusion

Our analysis of self-reported progress by national leadership indicates that community participation in MNH decision-making remains insufficiently embedded in health systems. While many LMICs report community participation during early policy cycle stages - in priority setting and planning - this involvement often diminishes during implementation where it is most needed to drive system-level change.

Substantial effort is required to institutionalise community participation across the entire MNH policy cycle, in line with government commitments to the 2024 World Health Assembly Resolutions for participation in decision-making (WHA77.2) and accelerating progress for maternal, newborn and child health (WHA77.5) [4,70]. Strengthening this integration will require clear metrics to track implementation, enabling more accurate assessments of progress and accountability.

Community participation was reported across diverse geographies, with the highest levels observed in Sub-Saharan Africa and South Asia. However, the variability across countries highlights the importance of exploring the context-specific drivers of progress. Understanding these drivers, including the technical, political, organisational, and resource factors that contribute to sustained and scalable participation, can help countries adapt and embed successful approaches within their health systems.

## Supporting information

S1 Table (XLSX)

S2 Table (XLSX)
